# Citric acid as a safe alternative to oxalic acid in the Gomori reticulin technique: a comparative study

**DOI:** 10.1007/s00418-025-02392-3

**Published:** 2025-06-24

**Authors:** Ana Bento, Ana Nascimento, Sofia Nobre, Teresa Ferreira, Amadeu Borges-Ferro, Ana Marques-Ramos

**Affiliations:** 1 Escola Superior de Tecnologia da Saúde de Lisboa, Instituto Politécnico de Lisboa (ESTeSL), Av. D. João II, Lote 4.69.01, 1990-096 Lisbon, Portugal; 2 Instituto Português de Oncologia Francisco Gentil, Lisbon, Portugal; 3 Health & Technology Research Center (H&TRC), Escola Superior de Tecnologia da Saúde, Instituto Politécnico de Lisboa (ESTeSL), Lisbon, Portugal

**Keywords:** Citric acid, Oxalic acid substitution, Gomori reticulin, Potassium permanganate

## Abstract

The use of oxalic acid is common in metallic impregnation techniques, where it serves as a reducing and bleaching agent for potassium permanganate. Given that oxalic acid is harmful to health, its replacement with another reagent is advantageous for healthcare professionals handling it in laboratory settings. Considering that citric acid can also reduce potassium permanganate, the aim of this study was to determine whether oxalic acid in the Gomori reticulin technique could be replaced by citric acid, a compound substantially less hazardous to health. To this end, 1%, 5%, and 10% citric acid solutions were tested on porcine liver and kidney samples, and the intensity of reticulin fiber staining, contrast, and overall morphological preservation were assessed in comparison with 1% oxalic acid. The results showed no statistically significant differences between the positive control (oxalic acid) and the citric acid protocols, with the 5% citric acid concentration proving most favorable in terms of required incubation time and the evaluated morphological parameters. It was therefore concluded that citric acid can be used as a bleaching agent for potassium permanganate in the Gomori technique, effectively replacing oxalic acid.

**Supplementary Information:**

The online version contains supplementary material available at 10.1007/s00418-025-02392-3.

## Introduction

Silver impregnation techniques for reticulin fiber visualization have evolved over the last two centuries. Early medical uses of silver nitrate revealed its affinity for reticulin fibers, and by 1905, Maresch had adapted silver methods for neurofibril identification (Little and Kramer 1952; Puchtler and Waldrop 1978; Javaeed et al. 2021). A major advancement came in 1937, when György Gömöri standardized the silver impregnation technique for reticulin fibers, establishing a protocol involving oxidation with potassium permanganate (KMnO_4_), followed by treatment with oxalic acid (H_2_C_2_O_4_), hydrobromic acid, or metabisulfite, before silver impregnation and reduction to metallic silver (Gömöri 1937). Of these sequential steps, oxalic acid is critical to reduce permanganate ions (MnO_4_^−^) to soluble Mn^2^^+^, thereby decolorizing the tissue and removing residual KMnO_4_. However, oxalic acid poses significant health risks due to its corrosive and toxic nature upon ingestion or inhalation (Noonan and Savage 1999; PubChem). In analytical experiments conducted by Berka et al., it was demonstrated that potassium permanganate oxidizes citric acid, resulting in the formation of carbon dioxide and water (Berka et al. 1979). Given citric acid’s substantially lower toxicity (PubChem), it emerges as a promising substitute for oxalic acid in the Gomori reticulin staining protocol. Hence, this study aimed to evaluate whether citric acid could effectively replace oxalic acid in the potassium permanganate bleaching step of the Gomori technique.

## Materials and methods

Kidney and liver samples were obtained from commercially sourced pork purchased at a local butcher shop. As the tissues were not collected from live animals specifically for this study, information such as species, age, sex, or use of anesthesia was not applicable. Following sample acquisition, macroscopic examination was performed by opening the kidneys along the greater curvature and slicing the livers. This allowed obtaining representative fragments of approximately 20 × 4 mm from the cortical and medullary zones and the hepatic parenchyma, respectively. The Gomori reticulin stain highlights the reticulin fiber network surrounding glomeruli, tubules, and blood vessels in the kidney (Gömöri 1937; Wheater and Burkitt 1997), and the reticulin fibers in the space of Disse *(*between hepatocyte plates and sinusoidal endothelial cells), enabling analysis of hepatocyte plate thickness in the liver (Gömöri 1937; Saxena 2018). Subsequently, samples were fixed in 10% formalin for 24 h at room temperature (23 ± 2 °C) and processed for histology. The fragments were then embedded in paraffin to form blocks and sectioned at 3 µm (μm) thickness, followed by histological section adhesion to slides for 1 h at 60 °C.

The protocol used for Gomori staining technique was adapted from the methods developed by Bielschowsky-Foot (Ebling 2022) and Gomori in 1937 (Gömöri 1937) (Table 1). The first step involves oxidation, where glycol groups of hexoses are oxidized to aldehydes by potassium permanganate action. Subsequently, in the bleaching step, excess potassium permanganate is removed with oxalic acid. This is followed by sensitization with a metallic salt, which enhances silver deposition and increases final contrast. Ferric ammonium sulfate acts as a mordant, creating binding sites for silver diamine in the ammoniacal solution. After impregnation with this solution, formaldehyde reduces silver ions (Ag^+^) to elemental silver (Ag^0^), precipitating metallic deposits. Unreacted silver is then removed by sodium thiosulfate (Gömöri 1937; Carson and Cappellano 2009; Suvarna et al. 2013; Kiernan 2015). Counterstaining for this technique is optional and can be performed using light green or Kernechtrot. If higher definition and better overall contrast are desired, a toning step may be carried out before adding sodium thiosulfate. This involves replacing metallic silver with metallic gold, which offers greater stability (Kiernan 2015) (Table 1).

### Gomori reticulin staining protocol

**Table:** Table 1 Gomori reticulin staining protocol

1	Deparaffinize in xylene	15 min
2	Hydrate through a descending alcohol series to distilled water	—
3	Acidified potassium permanganate 0.5%	5 min
4	Wash in distilled water	—
**5**	**Oxalic acid 1% or citric acid 1%, 5%, or 10%**	**Varies**
6	Wash in distilled water	—
7	Ferric alum	15 min
8	Wash in distilled water	—
9	Ammoniacal silver nitrate (filtered)	30 min
10	Wash in distilled water	—
11	Formalin 10%	1 min
12	Wash in distilled water	—
13	Sodium thiosulfate 2.5%	2 min
14	Wash in distilled water	—
15	Light green SF yellowish	2 min
16	Wash in distilled water	—
17	Dehydrate through a graded series of alcohols	—
18	Clear in xylene and mount	10 min

To minimize errors, all tissue samples were processed under identical conditions and by the same technicians. A total of 32 histological sections of swine liver and kidney were subjected to Gomori reticulin staining protocol (steps 1–4). After rinsing in distilled water (step 4), the slides were divided as follows and treated until bleaching was achieved:

- 8 slides in 1% oxalic acid (positive control)
- 8 slides in 1% citric acid
- 8 slides in 5% citric acid
- 8 slides in 10% citric acid

Following steps 6–18 of the protocol, the slides were evaluated by two observers using predefined scoring criteria (Table 2). Images were acquired using a Zeiss Axioskop 40 microscope equipped with Zeiss objective lenses, and captured with an iPhone X (12 megapixel dual rear camera) running iOS 16 as the acquisition software.

**Table 2:** Scoring criteria for slide quality assessment

	Score			
	0	1	2	3
Morphological preservation (A)	Morphology not preserved; evaluation not possible	Moderate loss of morphology; evaluation still possible	Mild loss of morphology; evaluation still possible	Morphology preserved
Intensity of reticulin fiber impregnation (B)	Absent	Weak intensity	Moderate intensity	Strong intensity
Contrast (C)	Staining deficiencies; evaluation not possible	Moderate staining deficiencies; evaluation still possible	Mild staining deficiencies; evaluation not compromised	Staining enhances visualization of fiber impregnation

The total score was calculated using the following formula:

$$Total \, Score = \left[ {\left( {Ax0,30} \right) + \left( {Bx0,60} \right) + \left( {Cx0,10} \right)} \right]x33,33$$

The final multiplication by 33.33 scales the score to a 0–100 range, facilitating reader comprehension and interpretation.

Following score assignment by evaluators, data were organized and analyzed using IBM SPSS Statistics (Statistical Package for the Social Sciences) software for statistical comparison.

### Ethical considerations

This study presented no ethical conflicts, as it used commercially obtained swine tissue samples solely for research purposes, with no occurrence of animal mistreatment. The researchers declare no conflicts of interest. The work was approved by the Ethics Committee of Lisbon’s School of Health Technology (ESTeSL; Authorization CE-ESTeSL No. 15–2023) and supported by the Portuguese Oncology Institute of Lisbon Francisco Gentil, which supplied all required materials and reagents.

## Results and discussion

### Assessment of citric acid as a safer alternative to oxalic acid in Gomori reticulin stain

To evaluate the feasibility of substituting oxalic acid (a compound with health risks) with the more inert citric acid in Gomori technique, we first analyzed reticular fiber staining using three different citric acid concentrations (1%, 5%, and 10%). The incubation time for each concentration was determined through macroscopic evaluation of potassium permanganate bleaching capacity. Results showed incubation times of 35 min (1% citric acid), 25 min (5%), and 20 min (10%), compared with 1.5 min for oxalic acid in the same processing step.

**Fig. 1:**
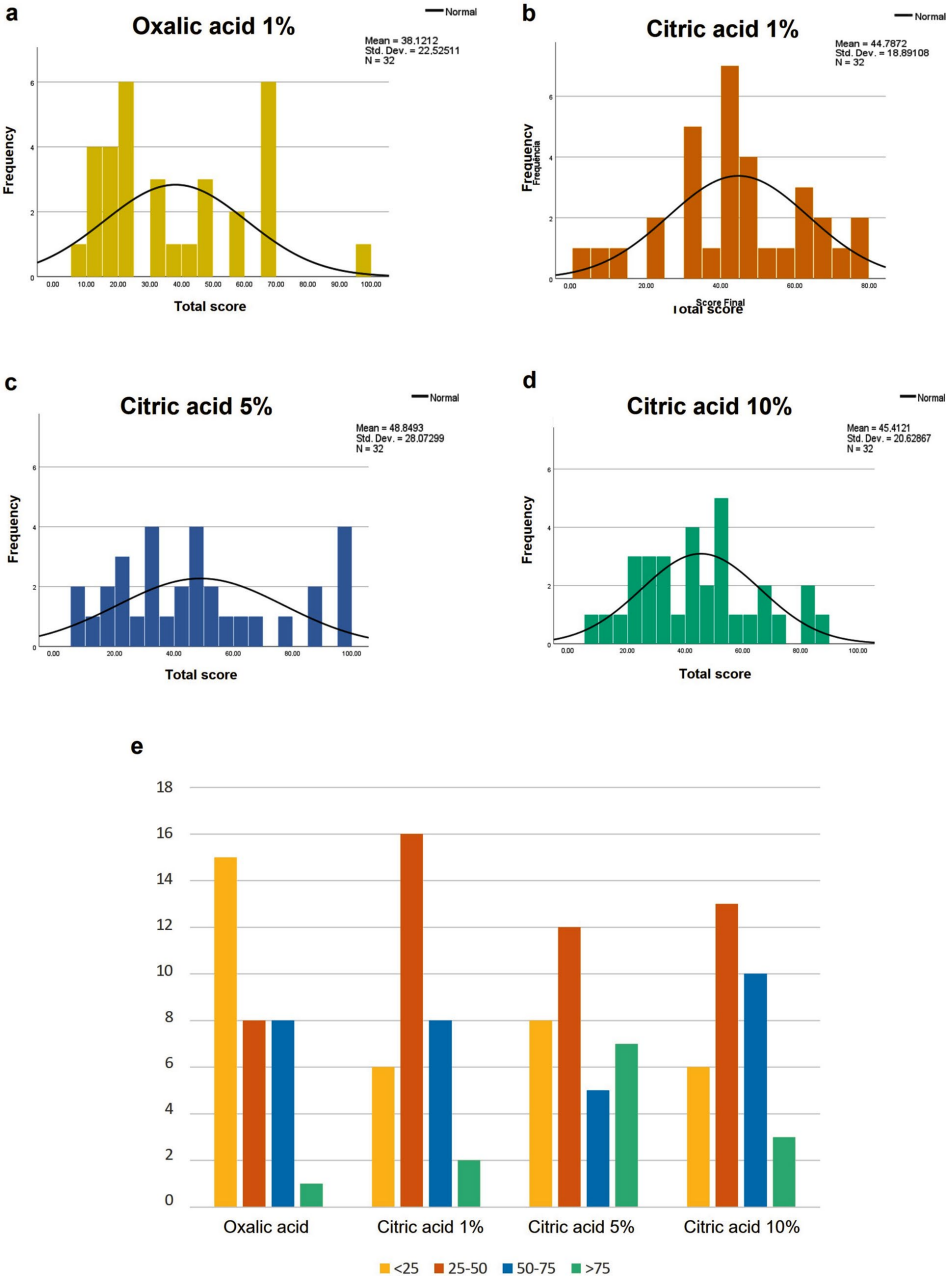
Frequency distribution of total scores in Gomori reticulin stain across different potassium permanganate bleaching conditions. **a** Oxalic acid (positive control); **b** 1% citric acid; **c** 5% citric acid; and **d** 10% citric acid. **e** Slide distribution by total score ranges

Microscopic examination of slides led evaluators to assign scores from 0 to 3 for the parameters listed in Table 2, which were then used to calculate each slide’s total score. The results showed considerable score dispersion across all tested conditions, including the standard oxalic acid protocol (Fig. 1; Supplementary Table 1). In the citric acid protocols (1%, 5%, and 10%), most slides achieved final scores between 25 and 50 (16, 12, and 13 slides, respectively), while with 1% oxalic acid the majority of slides scored below 25 (Fig. 1e). This higher score prevalence with citric acid conditions indicates they provide good reticular fiber staining. Notably, the 5% citric acid condition produced the highest number of maximum scores (seven slides, compared with just one to three slides in other conditions; Fig. 1e), suggesting that 5% citric acid may represent the optimal concentration for potassium permanganate bleaching in Gomori reticulin stain, offering a safer alternative without compromising staining quality.

### Comparative analysis of bleaching agents

**Fig. 2:**
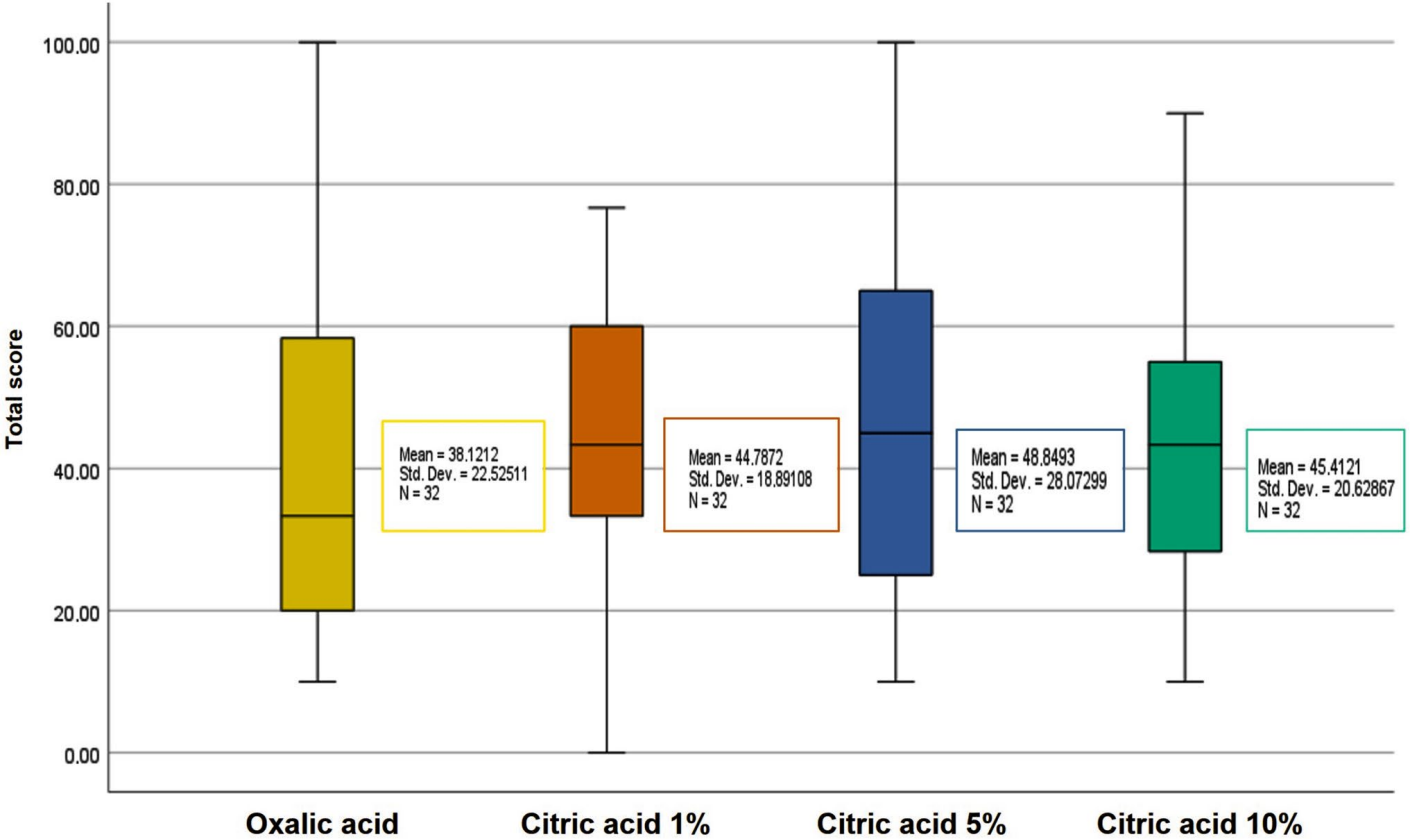
Comparison of mean total scores in Gomori reticulin stain using either oxalic acid (positive control) or citric acid (1%, 5%, and 10%) for potassium permanganate bleaching

When evaluating the mean scores across all four protocols, no pronounced differences were observed between test conditions. However, slides processed with citric acid protocols consistently showed higher final mean scores compared with the original oxalic acid protocol (Fig. 2; Supplementary Table 1). The highest mean score was achieved with 5% citric acid (49), followed by both 1% and 10% concentrations (45 each), while oxalic acid yielded the lowest mean score (38) (Fig. 2). These results confirm that citric acid, particularly at 5% concentration, serves as an effective bleaching agent in metal impregnation techniques.

Of note, while some citric-acid-treated slides showed complete absence of fiber staining, this phenomenon also occurred in positive controls (oxalic acid). This observation suggests that any staining failures were likely attributable to technical execution errors rather than the experimental variables under investigation.

### Evaluation of citric acid’s effects on Gomori reticulin stain

Subsequent analysis examined whether citric acid application in Gomori technique differentially impacted tissue morphology, reticular fiber staining, or observed contrast. The results revealed that 5% citric acid best preserved tissue morphology (score: 1.94), followed by 1% (1.88) and 10% (1.81) concentrations, while the positive control (oxalic acid) showed the lowest score (1.66). Representative images demonstrating these outcomes are presented in Fig. 4.

**Fig. 3:**
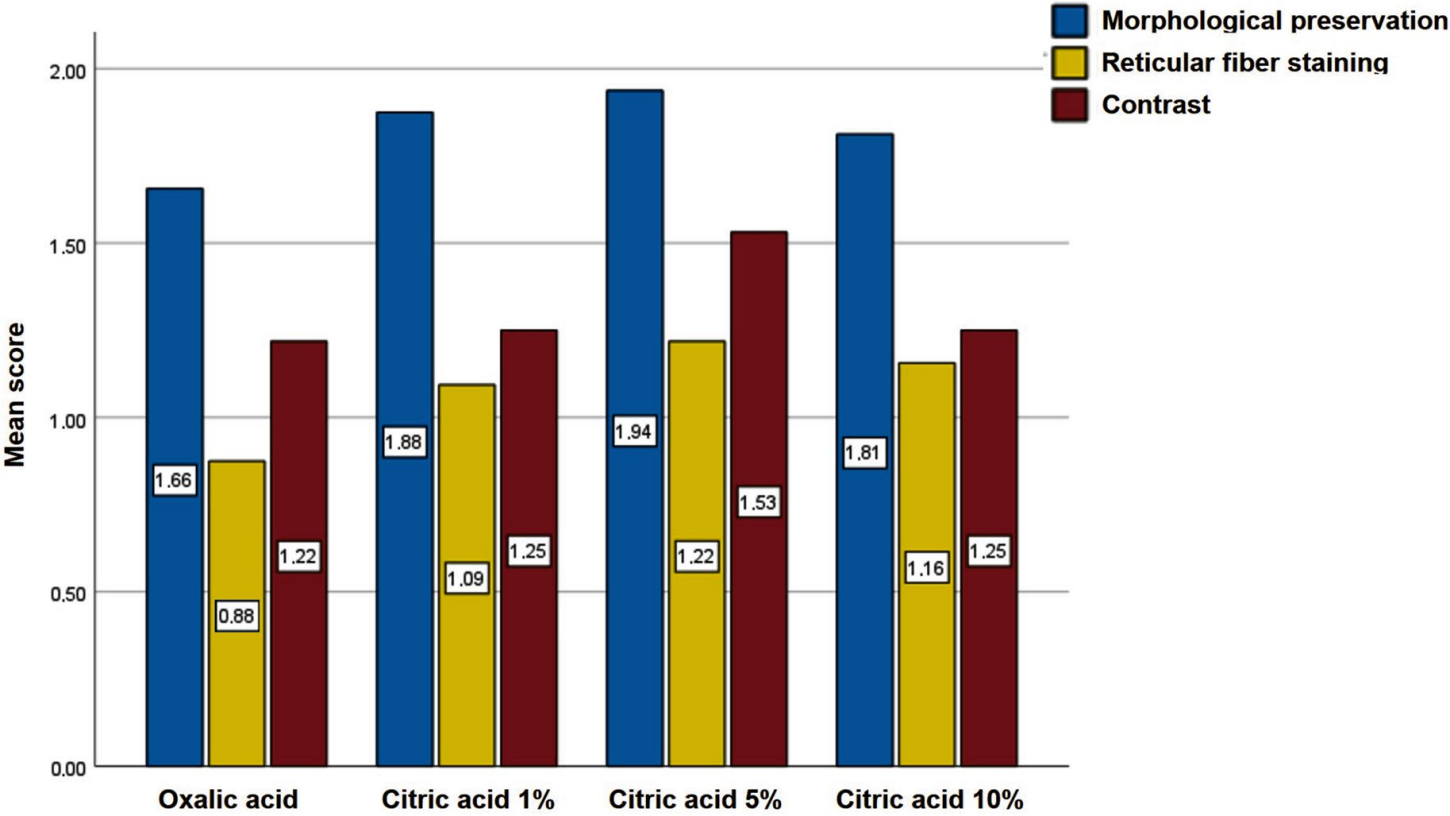
Scoring results for tissue morphology preservation, reticular fiber staining, and observed contrast in Gomori reticulin stain using either oxalic acid (positive control) or citric acid (1%, 5%, and 10%) for potassium permanganate bleaching

**Fig. 4:**
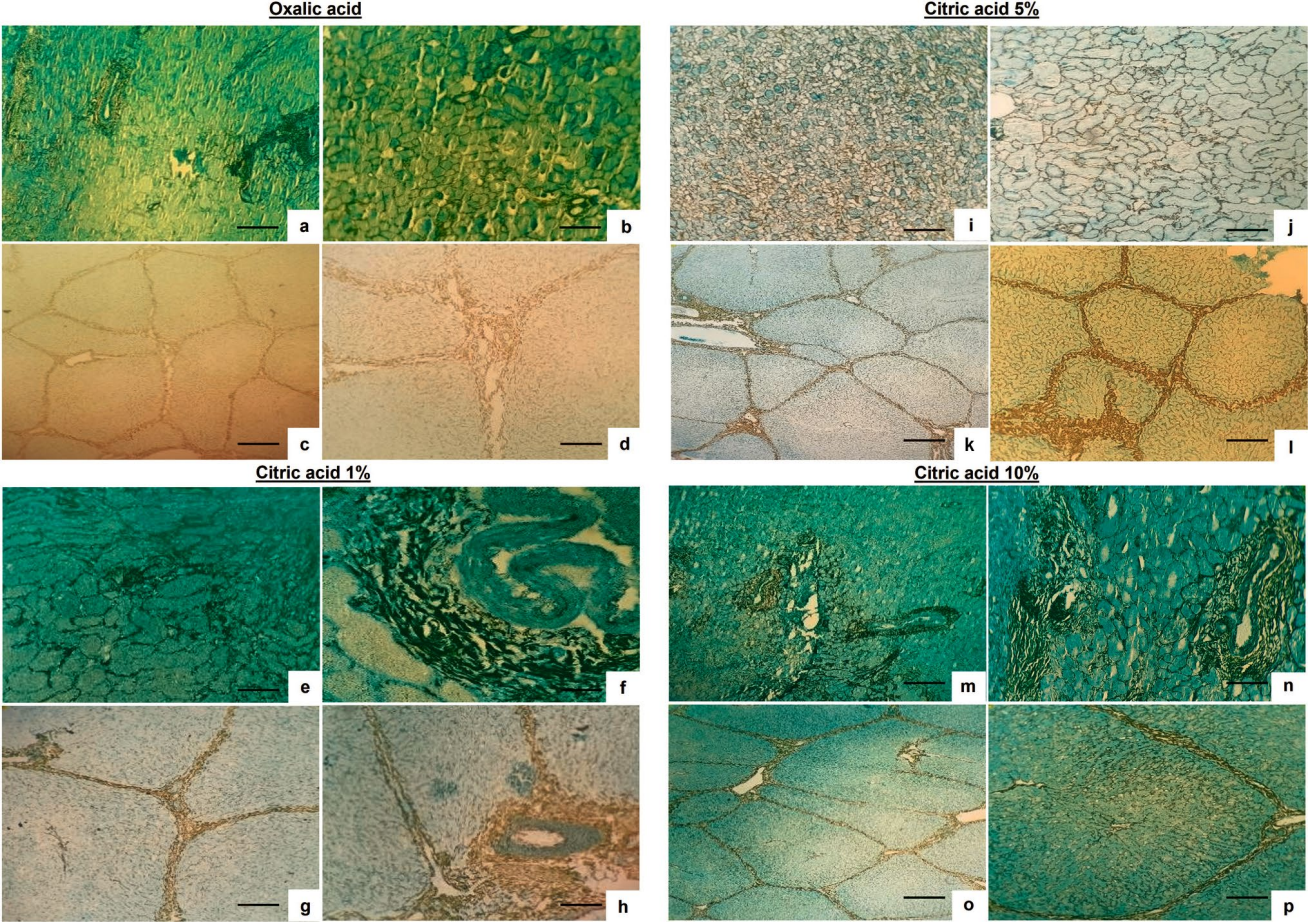
Reticulin fiber staining performed using Gomori method with light green counterstain. Potassium permanganate bleaching was carried out using the following agents: **a–d** Oxalic acid: **a–b** kidney (**a** scale bar = 25 μm; **b** scale bar = 10 μm) and **c–d** liver (**c** scale bar = 25 μm; **d** scale bar = 10 μm); **e–h** 1% citric acid: **e–f** kidney (**e** scale bar = 25 μm; **f** scale bar = 10 μm) and **g–h** liver (**g** scale bar = 25 μm; **h** scale bar = 10 μm); **i–l** 5% citric acid: **i–j** kidney (**i** scale bar = 25 μm; **j** scale bar = 10 μm) and " -->**k–l** liver (**k** scale bar = 25 μm; **l** scale bar = 10 μm); and **m–p** 10% citric acid: **m–n** kidney (**m** scale bar = 25 μm; **n** scale bar = 10 μm) and **o–p** liver (**o** scale bar = 25 μm; **p** scale bar = 10 μm)

Regarding staining contrast, 5% citric acid again produced the highest value (1.53), with both other concentrations scoring equally (1.25), compared with oxalic acid’s 1.22. For reticular fiber staining, 5% citric acid remained superior (1.22), followed by 10% (1.16) and 1% (1.09) concentrations, compared with oxalic acid’s 0.88. These findings collectively demonstrate that 5% citric acid represents the most favorable condition for potassium permanganate bleaching in Gomori reticulin stain, providing optimal results not only for reticular fiber staining but also for contrast quality and overall tissue architecture preservation. Figure 3 presents the quantitative comparison of mean scores for each evaluated parameter, while visual evidence supporting these findings is shown in Fig. 4

This comprehensive evaluation establishes 5% citric acid as an effective alternative to oxalic acid, addressing safety concerns while maintaining or improving technical performance across all evaluated parameters of the staining technique.

### Statistical validation

To validate our experimental observations, we performed a detailed statistical analysis of the results (Supplementary Fig. 1). The Shapiro–Wilk normality test indicated normal distribution for both 1% citric acid (*W* = 0.963; *p* = 0.340) and 10% citric acid (*W* = 0.968; *p* = 0.457), while revealing non-normal distribution for oxalic acid (*W* = 0.891; *p* = 0.004) and 5% citric acid (W = 0.916; *p* = 0.016) (Supplementary Fig. 1a). Although these normality assumptions were partially violated, the subsequent Kruskal–Wallis non-parametric test (Supplementary Fig. 1b) demonstrated no statistically significant differences between the different protocol conditions (*p* = 0.324). The high degree of interobserver agreement was further confirmed by an excellent Cronbach’s alpha coefficient (*α* = 0.870; Supplementary Fig. 1c), indicating strong consistency between evaluators.

Importantly, despite this statistical similarity across conditions, our comprehensive morphological analysis consistently identified qualitative advantages when using 5% citric acid, particularly in three key aspects: superior reticular fiber staining quality, enhanced tissue contrast visualization, and optimal preservation of tissue architecture. Based on these combined statistical and morphological findings, we conclude that citric acid represents a viable and effective substitute for oxalic acid in the potassium permanganate bleaching step of Gomori reticulin stain, with the 5% concentration emerging as the most favorable and recommended formulation for routine laboratory use.

The silver impregnation techniques were originally established by Gomori in 1937 (Gömöri 1937), employing potassium permanganate oxidation as the initial step. During this phase, hydroxyl groups (-OH) from hexoses present in reticulin fibers undergo chemical conversion to aldehyde groups (-CHO). This molecular transformation reveals the fibers’ argyrophilic properties, enabling their characteristic black staining through subsequent silver impregnation (Kiernan 2015). After this oxidative process, the removal of the potassium permanganate excess and nonspecific staining residues is required through treatment with oxalic acid, hydrobromic acid, or metabisulfite.

In the Bielschowsky-Foot protocol, oxalic acid serves as a reducing agent in the post-oxidative step, converting residual MnO4^−^ into colorless Mn^2+^ while eliminating potassium permanganate staining artifacts (Ebling 2022). Although typically used in low concentrations in histological protocols, oxalic acid remains a hazardous substance, particularly in cases of repeated or unprotected exposure. It is highly toxic and corrosive, with ingestion or inhalation potentially causing conjunctivitis, skin ulcerations (including gangrenous lesions), respiratory tract irritation, mucosal damage, headache, and generalized weakness (PubChem). Its metabolic toxicity results primarily from the formation of insoluble calcium oxalate crystals, disrupting calcium–potassium homeostasis and potentially leading to renal calculi and intestinal irritation. Additionally, oxalic acid chelates iron, impairing physiological oxygen transport (PubChem). According to the Hommel diagram, it carries a health hazard rating of 3 (on a scale of 0–4), indicating potential for severe or permanent injury in emergency exposures (PubChem).

In contrast, citric acid—a naturally occurring tricarboxylic acid in citrus fruits—demonstrates low acute toxicity. The Hommel diagram assigns it a hazard rating of 1, indicating only minor residual health effects under accidental exposure conditions (PubChem). In 1917, Dhar first demonstrated that carboxylic acids—including citric acid—undergo oxidation reactions with potassium permanganate. Berka et al. (Berka et al. 1979) subsequently confirmed the reaction stoichiometry:

$$6KMnO_{4} + C_{6} H_{8} O_{7} \to 6MnO_{2} + 6CO_{2} + 4H_{2} O + 3K_{2} O$$

Thermodynamically, citric acid functions as a reducing agent analogous to oxalic acid, though with distinct kinetic and mechanistic properties (PubChem). Building upon these chemical principles, we hypothesized that citric acid could effectively replace oxalic acid in Gomori reticulin method, serving as a safer yet equally efficient bleaching agent for removing excess KMnO_4_ from histological sections. This substitution offers significant advantages given citric acid’s substantially lower toxicity profile compared with oxalic acid, while maintaining comparable bleaching efficacy in tissue processing. In fact, the results obtained in the present study indicate that citric acid is an adequate substitute for oxalic acid in the Gomori technique. Although no statistically significant differences were found, the use of citric acid allows for better staining of reticulin fibers, greater morphological preservation, and higher contrast between the fibers and adjacent structures (Fig. 3). Thus, citric acid proves to be a promising reducing agent for potassium permanganate, particularly at a concentration of 5%.

The experimental results demonstrated that the reaction time required for citric acid at all tested concentrations was longer than that observed for oxalic acid. This kinetic difference can be explained by the stoichiometric and mechanistic characteristics of the respective oxidation reactions. While the complete oxidation of citric acid (C_6_H_8_O_7_) requires six molecules of KMnO_4_ (6 KMnO_4_ + C_6_H_8_O_7_ → 6 MnO_2_ + 6 CO_2_ + 4 H_2_O + 3 K_2_O), oxalic acid (H_2_C_2_O_4_) has a more favorable stoichiometry (2 KMnO_4_ + 3 H_2_C_2_O_4_ → 2 MnO_2_ + 6 CO_2_ + 4 H_2_O + K_2_O), consuming proportionally less permanganate. Additionally, the tricarboxylic structure of citric acid, more complex than the dicarboxylic structure of oxalic acid, involves a gradual oxidation mechanism in multiple stages. These combined factors—higher oxidant consumption per substrate molecule and greater structural complexity—may quantitatively justify the longer reaction times observed for citric acid under equivalent experimental conditions.

Thus, the substitution of oxalic acid by citric acid in the Gomori technique results in a more time-consuming process. To address this issue and make the technique faster, the incubation with citric acid could be performed using a microwave oven. The use of microwave ovens in histopathology has been explored since the 1970s, as microwaves accelerate reactions by increasing molecular kinetic energy (Priya et al. 2020). This principle, when applied to the Gomori technique using citric acid, would enable faster penetration of the acid molecules, resulting in a reduction of the overall technique time. Some studies indicate that the results obtained with the use of microwaves in histochemical techniques showed no significant changes in cytoplasmic and nuclear staining, nuclear contour and chromatin, or staining intensity (Mukunda et al. 2015; Priya et al. 2020). Thus, it will become feasible to use microwave ovens to make the Gomori reticulin technique not only safer by eliminating exposure to oxalic acid, but also faster.

Silver impregnation techniques are essential histopathological tools for evaluating tissue architecture in various organs, with particular relevance in the diagnosis of malignant neoplasms (Dey 2018; Venkatesh and Malaichamy 2019). In the liver, they allow for the precise identification of fibrogenic patterns (fibrosis and cirrhosis) and the detection of critical structural alterations, such as collapse or condensation of reticulin fibers—histological markers of loss of hepatic parenchyma (Krishna 2013). In the kidney, these techniques are indispensable for staging glomerulopathies, particularly in the characterization of proliferative membranous glomerulonephritis and membranous glomerulopathy (Windrum et al. 1955; Cathro et al. 2018). Given their diagnostic importance, the continuous improvement of these methods is crucial, aiming not only at optimizing histomorphological results but also ensuring laboratory safety during their execution.

## Supplementary Information

Below is the link to the electronic supplementary material.

Supplementary file1 (DOCX 107 KB)

Supplementary file2 (DOCX 16 KB)

## Data Availability

Data are provided within the manuscript or supplementary information files.

## References

[CR1] Berka A, Barek J, Hladíková A (1979) Analysis of mixtures of citric and oxalic acids based on their oxidation with potassium permanganate and manganese(III) sulfate. Microchem J 24:431–434. 10.1016/0026-265X(79)90087-0

[CR2] Carson FL, Cappellano CH (2009) Histotechnology: a self-instructional text. ASCP Press, Chicago

[CR3] Cathro HP, Shen SS, Truong LD (2018) Diagnostic histochemistry in medical diseases of the kidney. Semin Diagn Pathol 35:360–369. 10.1053/j.semdp.2018.10.001 30366793 10.1053/j.semdp.2018.10.001

[CR4] Dey P (2018) Basic and advanced laboratory techniques in histopathology and cytology. Singapore, Springer Singapore, p 345

[CR5] Ebling H (2022) Coloração de fibras de reticulina em material calcificado. Rev Fac Odontol Porto Alegre 2:43–49. 10.22456/2177-0018.127109

[CR6] Gömöri G (1937) Silver impregnation of reticulum in paraffin sections. Am J Pathol 13(993–1002):5 PMC1911151 19970363

[CR7] Javaeed A, Qamar S, Ali S et al (2021) Histological stains in the past, present, and future. Cureus. 10.7759/cureus.18486 34754648 10.7759/cureus.18486 PMC8566793

[CR8] Kiernan JA (2015) Histological and histochemical methods: theory and practice. Scion Publishing Ltd., UK, pp 419–453

[CR9] Krishna M (2013) Role of special stains in diagnostic liver pathology. Clin Liver Dis 2:S8–S10. 10.1002/cld.148 10.1002/cld.148 PMC6448668 30992876

[CR10] Little K, Kramer H (1952) Nature of reticulin. Nature 170:499–500. 10.1038/170499b0 12993215 10.1038/170499b0

[CR11] Mukunda A, Narayan T, Shreedhar B et al (2015) Accelerated staining technique using kitchen microwave oven. Indian J Pathol Microbiol 58:316. 10.4103/0377-4929.162863 26275253 10.4103/0377-4929.162863

[CR12] Noonan SC, Savage GP (1999) Oxalate content of foods and its effect on humans. Asia Pac J Clin Nutr 8:64–74 24393738

[CR13] Priya AHh, Vb V, Chellaswamy S et al (2020) Evaluation of efficacy of microwave staining over conventional staining in replicating tissue architecture: a prospective study. J Pharm Bioallied Sci 12:283. 10.4103/jpbs.JPBS_86_20 10.4103/jpbs.JPBS_86_20 PMC7595548 33149472

[CR14] PubChem Citric Acid. https://pubchem.ncbi.nlm.nih.gov/compound/311. Accessed 7 Apr 2025b

[CR15] PubChem Oxalic Acid. https://pubchem.ncbi.nlm.nih.gov/compound/971. Accessed 7 Apr 2025a

[CR16] Puchtler H, Waldrop FS (1978) Silver impregnation methods for reticulum fibers and reticulin: a re-investigation of their origins and specifity. Histochemistry 57:177–187. 10.1007/BF00492078 711512 10.1007/BF00492078

[CR17] Saxena R (2018) Microscopic anatomy, basic terms, and elemental lesions. Practical hepatic pathology: a diagnostic approach. Elsevier, Spain, pp 3–29

[CR18] Suvarna KS, Layton C, Bancroft JD (2013) Theory and practice of histological techniques, 7th edn. Elsevier Churchill Livingston, Edinburgh

[CR19] Venkatesh V, Malaichamy V (2019) Role of special stains as a useful complementary tool in the diagnosis of renal diseases: a case series study. Int J Res Med Sci 7:1539. 10.18203/2320-6012.ijrms20191632

[CR20] Wheater PR, Burkitt HG (1997) Wheater’s functional histology: a text and colour atlas, 3rd edn. Churchill Livingstone, Edinburgh, p 45

[CR21] Windrum GM, Kent PW, Eastoe JE (1955) The constitution of human renal reticulin. Br J Exp Pathol 36:49–59 14351637 PMC2082517

