# Supplementary material for: Citric acid as a safe alternative to oxalic acid in the Gomori reticulin technique: a comparative study
Source: Histochem Cell Biol. 2025 Jun 24;163(1):69. doi: 10.1007/s00418-025-02392-3 (PMC12187824; doi:10.1007/s00418-025-02392-3)

Citric Acid as a Safe Alternative to Oxalic Acid in the Gomori Reticulin Technique: A Comparative Study

Ana Bento1,^φ^, Ana Nascimento1,^φ^, Sofia Nobre1,^φ^, Teresa Ferreira2, Amadeu Borges-Ferro1, Ana Marques-Ramos1,3,*

**Histochemistry and Cell Biology**

**Supplementary Figure 1 –** Statistical analysis: (**a**) Shapiro–Wilk test for normality; (**b**) Non-parametric test for comparison of total score means; (**c**) Comparison of the mean total scores assigned by the two evaluators using Cronbach’s alpha.

1. The significance level is ,050.
2. Asymptotic significance is displayed.

**b**


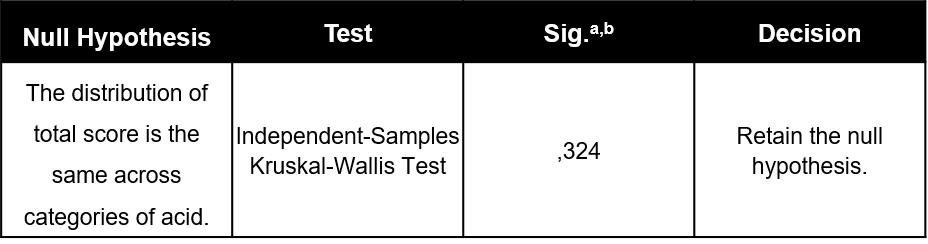

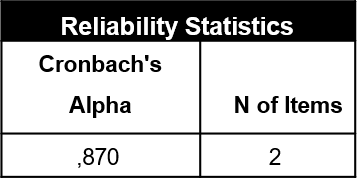


**c**

**a**


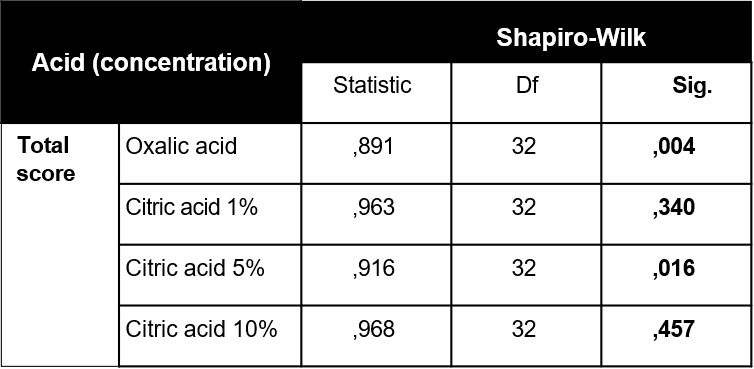

Supplement: Supplementary file 1 — Supplementary file1 (DOCX 107 KB) [file 418_2025_2392_MOESM1_ESM.docx]
