# Supplementary material for: Citric acid as a safe alternative to oxalic acid in the Gomori reticulin technique: a comparative study
Source: Histochem Cell Biol. 2025 Jun 24;163(1):69. doi: 10.1007/s00418-025-02392-3 (PMC12187824; doi:10.1007/s00418-025-02392-3)
Supplement: Supplementary file 2 — Supplementary file2 (DOCX 16 KB) [file 418_2025_2392_MOESM2_ESM.docx]

Citric Acid as a Safe Alternative to Oxalic Acid in the Gomori Reticulin Technique: A Comparative Study

Ana Bento1,^φ^, Ana Nascimento1,^φ^, Sofia Nobre1,^φ^, Teresa Ferreira2, Amadeu Borges-Ferro1, Ana Marques-Ramos1,3,*

**Histochemistry and Cell Biology**

**Supplementary Table 1** – Descriptive statistical analysis of the Final Score results, discriminated by differentiating acid

|  | *Oxalic acid 1%* | *Citric acid 1%* | *Citric acid 5%* | *Citric acid 10%* |
| --- | --- | --- | --- | --- |
| Mean | 38,12119 | 44,78719 | 48,84928 | 45,41213 |
| Standard Error | 3,981915 | 3,339503 | 4,962651 | 3,646669 |
| Median | 33,33 | 43,329 | 44,9955 | 43,329 |
| Mode | 23,331 | 43,329 | 33,33 | 53,328 |
| Standard Deviation | 22,52511 | 18,89108 | 28,07299 | 20,62867 |
| Sample variance | 507,3806 | 356,8731 | 788,0928 | 425,5421 |
| Kurtosis | 0,009734 | -0,01874 | -0,72616 | -0,42272 |
| Skewness | 0,808071 | -0,27775 | 0,599931 | 0,389186 |
| Range | 89,991 | 76,659 | 89,991 | 79,992 |
| Minimum | 9,999 | 0 | 9,999 | 9,999 |
| Maximum | 99,99 | 76,659 | 99,99 | 89,991 |
| Sum | 1219,878 | 1433,19 | 1563,177 | 1453,188 |
| Count | 32 | 32 | 32 | 32 |
